# Commercial Immunoglobulin Products Contain Neutralizing Antibodies Against Severe Acute Respiratory Syndrome Coronavirus 2 Spike Protein

**DOI:** 10.1093/cid/ciad368

**Published:** 2023-06-20

**Authors:** Vinit Upasani, Katie Townsend, Mary Y Wu, Edward J Carr, Agnieszka Hobbs, Giulia Dowgier, Martina Ragno, Lou S Herman, Sonal Sharma, Devesh Shah, Simon F K Lee, Neil Chauhan, Julie M Glanville, Lucy Neave, Steven Hanson, Sriram Ravichandran, Aoife Tynan, Mary O’Sullivan, Fernando Moreira, Sarita Workman, Andrew Symes, Siobhan O Burns, Susan Tadros, Jennifer C L Hart, Rupert C L Beale, Sonia Gandhi, Emma C Wall, Laura McCoy, David M Lowe

**Affiliations:** Institute of Immunity and Transplantation, University College London (UCL), London, United Kingdom; Department of Clinical Immunology, Royal Free London National Health Service (NHS) Foundation Trust, London, United Kingdom; COVID Surveillance Unit, Francis Crick Institute, London, United Kingdom; Francis Crick Institute, London, United Kingdom; Department of Renal Medicine, Royal Free London NHS Foundation Trust, London, United Kingdom; COVID Surveillance Unit, Francis Crick Institute, London, United Kingdom; COVID Surveillance Unit, Francis Crick Institute, London, United Kingdom; COVID Surveillance Unit, Francis Crick Institute, London, United Kingdom; COVID Surveillance Unit, Francis Crick Institute, London, United Kingdom; Department of Elderly Medicine, Barnet Hospital, Royal Free London NHS Foundation Trust, London, United Kingdom; Department of Elderly Medicine, Barnet Hospital, Royal Free London NHS Foundation Trust, London, United Kingdom; Department of Infectious Diseases, Royal Free London NHS Foundation Trust, London, United Kingdom; Department of Haematology, Royal Free London NHS Foundation Trust, London, United Kingdom; Department of Haematology, Royal Free London NHS Foundation Trust, London, United Kingdom; Department of Haematology, Royal Free London NHS Foundation Trust, London, United Kingdom; Department of Haematology, Royal Free London NHS Foundation Trust, London, United Kingdom; Department of Haematology, Royal Free London NHS Foundation Trust, London, United Kingdom; Department of Pharmacy, Royal Free London NHS Foundation Trust, London, United Kingdom; Department of Clinical Immunology, Royal Free London National Health Service (NHS) Foundation Trust, London, United Kingdom; Department of Clinical Immunology, Royal Free London National Health Service (NHS) Foundation Trust, London, United Kingdom; Department of Clinical Immunology, Royal Free London National Health Service (NHS) Foundation Trust, London, United Kingdom; Department of Clinical Immunology, Royal Free London National Health Service (NHS) Foundation Trust, London, United Kingdom; Institute of Immunity and Transplantation, University College London (UCL), London, United Kingdom; Department of Clinical Immunology, Royal Free London National Health Service (NHS) Foundation Trust, London, United Kingdom; Department of Clinical Immunology, Royal Free London National Health Service (NHS) Foundation Trust, London, United Kingdom; Department of Virology, Royal Free London NHS Foundation Trust, London, United Kingdom; COVID Surveillance Unit, Francis Crick Institute, London, United Kingdom; Department of Renal Medicine, Royal Free London NHS Foundation Trust, London, United Kingdom; COVID Surveillance Unit, Francis Crick Institute, London, United Kingdom; UCL Hospitals Biomedical Research Centre, London, United Kingdom; COVID Surveillance Unit, Francis Crick Institute, London, United Kingdom; UCL Hospitals Biomedical Research Centre, London, United Kingdom; Institute of Immunity and Transplantation, University College London (UCL), London, United Kingdom; Institute of Immunity and Transplantation, University College London (UCL), London, United Kingdom; Department of Clinical Immunology, Royal Free London National Health Service (NHS) Foundation Trust, London, United Kingdom

**Keywords:** SARS-CoV-2, spike antibody, IVIG, neutralization, immunodeficiency

## Abstract

**Background:**

Patients with antibody deficiency respond poorly to coronavirus disease 2019 (COVID-19) vaccination and are at risk of severe or prolonged infection. They are given long-term immunoglobulin replacement therapy (IRT) prepared from healthy donor plasma to confer passive immunity against infection. Following widespread COVID-19 vaccination alongside natural exposure, we hypothesized that immunoglobulin preparations will now contain neutralizing severe acute respiratory syndrome coronavirus 2 (SARS-CoV-2) spike antibodies, which confer protection against COVID-19 disease and may help to treat chronic infection.

**Methods:**

We evaluated anti–SARS-CoV-2 spike antibody in a cohort of patients before and after immunoglobulin infusion. Neutralizing capacity of patient samples and immunoglobulin products was assessed using in vitro pseudovirus and live-virus neutralization assays, the latter investigating multiple batches against current circulating Omicron variants. We describe the clinical course of 9 patients started on IRT during treatment of COVID-19.

**Results:**

In 35 individuals with antibody deficiency established on IRT, median anti-spike antibody titer increased from 2123 to 10 600 U/mL postinfusion, with corresponding increase in pseudovirus neutralization titers to levels comparable to healthy donors. Testing immunoglobulin products directly in the live-virus assay confirmed neutralization, including of BQ1.1 and XBB variants, but with variation between immunoglobulin products and batches.

Initiation of IRT alongside remdesivir in patients with antibody deficiency and prolonged COVID-19 infection (median 189 days, maximum >900 days with an ancestral viral strain) resulted in clearance of SARS-CoV-2 at a median of 20 days.

**Conclusions:**

Immunoglobulin preparations now contain neutralizing anti–SARS-CoV-2 antibodies that are transmitted to patients and help to treat COVID-19 in individuals with failure of humoral immunity.

## Teaser

Anti–severe acute respiratory syndrome coronavirus 2 spike antibodies with neutralizing activity against current variants are detectable in intravenous immunoglobulin (IVIG) products and transmitted to patients, whose serum demonstrates significantly improved viral neutralization capacity postinfusion. IVIG, together with remdesivir, successfully treats chronic coronavirus disease 2019.

**(See the Editorial Commentary by Senefeld and Joyner on pages 961–3.)**

Primary and secondary antibody deficiencies are characterized by impaired ability to mount a functional humoral immune response [1]. Patients with these conditions have either significantly decreased or no antibody responses after most infections or vaccinations compared to healthy individuals [1], including to severe acute respiratory syndrome coronavirus 2 (SARS-CoV-2) [2, 3]. These patients rely on long-term infusions with commercial immunoglobulin products, which may be administered subcutaneously or intravenously (IVIG), for protection against many infectious diseases. The products used for immunoglobulin replacement therapy (IRT) are derived from the plasma of thousands of prescreened healthy donors in the United States or mainland Europe containing antibodies against various antigens.

Since the beginning of the SARS-CoV-2 pandemic, individuals with antibody deficiencies have been at considerable risk of infection and have shown increased hospitalization rates and mortality [2, 4]. They are also at risk of prolonged and relapsing coronavirus disease 2019 (COVID-19), especially in the absence of B cells due to genetic disorders or B-cell–depleting therapies [3]. For individuals lacking a robust humoral vaccination response, attention has been given recently to other potential prophylactic strategies, such as long-acting monoclonal antibodies against the spike (S) protein of SARS-CoV-2 [5]. We hypothesized that, given the ubiquity of vaccination and/or SARS-CoV-2 infection even by 2021, immunoglobulin preparations prepared from plasma of healthy donors may now contain detectable levels of SARS-CoV-2 spike antibody. The products may therefore confer some protection, reducing the imperative for other prophylaxis, and may be useful adjuncts for treatment in patients unable to clear the infection, as has been proposed for convalescent plasma [6, 7].

Therefore, in this study, we strove to demonstrate the therapeutic potential of commercial IVIG products in COVID-19 using several approaches. First, we evaluated the antibody response against SARS-CoV-2 in a cohort of patients before and after undergoing IVIG infusion. Second, we estimated the neutralizing antibody titers against SARS-CoV-2 in these paired patient samples and the products themselves using an in vitro luminescence-based neutralization assay. Third, we tested the products against current circulating viral variants in a live-virus neutralization assay. Finally, we assessed the initiation of IVIG replacement during treatment of patients with antibody deficiency and chronic, relapsing COVID-19 or at high risk of persistent infection.

## METHODS

For the assessment of serum antibody titers, we recruited 35 individuals with immunodeficiencies on IRT and 7 healthy controls in July and August 2022. All donors provided written informed consent under protocols approved by National Health Service Research ethics committees (REC 04/Q0501/119 and 08/H0720/46). Blood sampling was performed from the patients in the clinic before and immediately after an IVIG infusion was completed. Patients at our center receive a range of immunoglobulin products with no particular preference, unless there are specific contraindications or adverse reactions.

We also present clinical data from 9 immunosuppressed patients with chronic/persistent polymerase chain reaction (PCR)–positive SARS-CoV-2 infection who received immunoglobulin therapy during their treatment for COVID-19. Written consent was obtained from all patients for their cases to be reported.

We determined the titers of antibodies directed against SARS-CoV-2 spike protein using a commercial immunoassay (Elecsys Anti–SARS-CoV-2 S, Roche). Samples with titers >2500 U/mL were diluted 10-fold and repeated, resulting in a maximum titer of 25 000 U/mL.

Neutralizing antibody titers against ancestral and Omicron BA.1 SARS-CoV-2 were determined using a luminescence-based pseudovirus neutralization assay as described previously [8]. In brief, serially diluted serum samples or neat immunoglobulin products were incubated in a 96-well plate with a human immunodeficiency virus–based pseudovirus expressing S protein of SARS-CoV-2 (ancestral or Omicron BA.1) on its surface. HeLa cells engineered to express ACE2, a surface attachment factor for SARS-CoV-2 [9], were added to the respective wells. After 3 days of incubation, cells were lysed, and plates were read upon addition of a luminescent substrate. Neutralization capacity of the serum sample is expressed in terms of inhibitory dilution 50 (ID_50_), that is, the serum dilution at which 50% of infection is inhibited compared to the virus alone.

IVIG products (Flebogamma, Gammaplex, Intratect, Iqymune, Octagam, and Privigen) were also tested in a live-virus microneutralization assay developed at the Francis Crick Institute against current Omicron variants of SARS-CoV-2 (Supplementary Table 1) as previously described [10]. In summary, serial dilutions of IVIG products were added to Vero E6 (Institut Pasteur) cells at 90% confluency before infection with live SARS-CoV-2 variants in 384-well format. Cells were fixed at a final formaldehyde concentration of 4% 24 hours after infection, permeabilized and blocked with a 3% BSA + 0.2% TritonX-100 solution in phosphate-buffered saline (v/v), and stained with DAPI to detect cellular nuclei and a Biotin-conjugated CR3009 antibody (produced in-house) with Alexa488-streptavidin (Invitrogen S32354) to detect infected cells expressing viral nucleoprotein. Whole-well images were captured at 5× on an Opera Phenix (PerkinElmer) and fluorescent areas calculated using the associated software Harmony (PerkinElmer). Infection per well is estimated as the measured area of green (Alexa488) divided by the measured area of blue (DAPI) before normalizing against the virus-only control wells and expressing as percentage of maximal infection. Data analysis was carried out in R. For each IVIG dilution series, a 4-parameter fit was modeled using drm from the drc package with some adjustments: lower limit = 0; upper limit = 110; Hill slope limits = 0.1–1.5.

## RESULTS

### SARS-CoV-2 Spike Antibody Titer and Neutralization Capacity of Serum Increase After IVIG Infusion

Patient demographic data for the cross-sectional study (n = 35) are summarized in Table 1. The most common diagnoses were common variable immunodeficiency and secondary hypogammaglobulinemia. All patients had been on regular IVIG for at least 6 months, and immunoglobulin G (IgG) trough levels were generally >7 g/L. All patients had received at least 2 vaccinations against SARS-CoV-2. Twenty patients reported a prior history of COVID-19 (2 patients reported 2 infections) with around half of episodes treated and others usually resolving spontaneously. Of note, 1 patient had received sotrovimab treatment within the previous 2 weeks.

**Table 1.** Patient Demographics and Clinical Details for Cross-sectional Study

Characteristic	No.
Age, y	
Median (range)	60 (20–86)
Sex	
Male	12
Female	23
Diagnosis	
CVID	12
XLA	2
Other/undefined primary hypogammaglobulinemia	9
Secondary hypogammaglobulinemia	12
Duration on IVIG	
6 mo–1 y	1
1–3 y	4
>3 y	30
Infusion frequency	
Every 2 wk	1
Every 3 wk	4
Every 4 wk	18
Every 5 wk	3
Every 6 wk	9
Last IgG trough, g/L	
Median (range)	9.3 (4.1–13.7)
Received COVID-19 vaccination	
No	0
Yes	35
2 vaccinations	3
3 vaccinations	7
4 vaccinations	19
5 vaccinations	6
Time since last vaccination, d	
Median (range)	124 (27–410)
Known previous COVID-19	
No	15
Yes, once	18
Yes, twice	2
COVID-19 episodes	
Untreated	10
Sotrovimab treatment	5
Remdesivir treatment	1
Nirmatrelvir/ritonavir treatment	1
Unknown treatment	5
Time since COVID-19, d	
Median (range)	131 (9–857)

Abbreviations: COVID-19, coronavirus disease 2019; CVID, common variable immunodeficiency; IgG, immunoglobulin G; IVIG, intravenous immunoglobulin; XLA, X-linked agammaglobulinemia.

**Figure 1.**
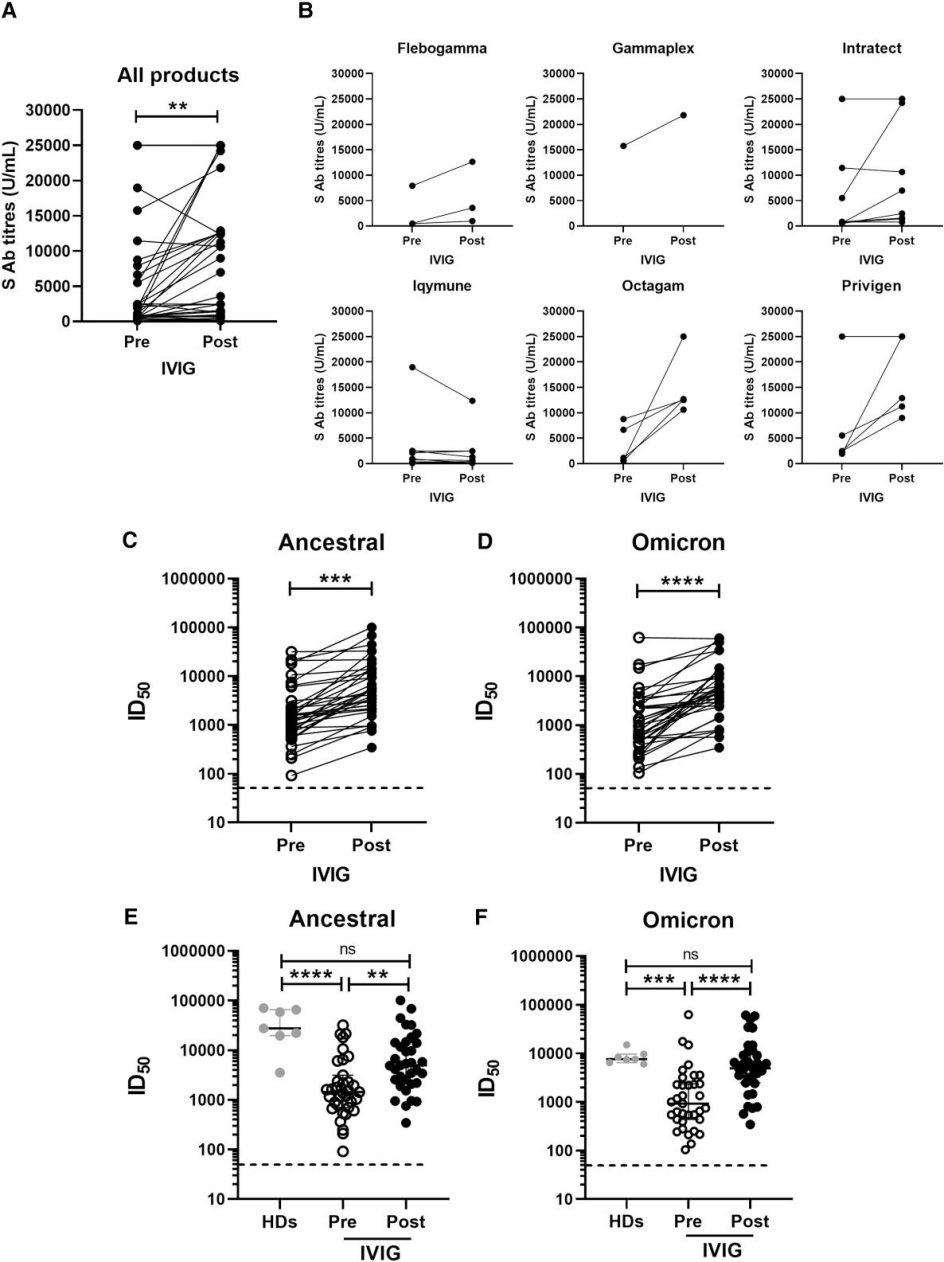
Humoral immune responses in patients with primary immunodeficiencies receiving intravenous immunoglobulin (IVIG) products. *A–B*, Severe acute respiratory syndrome coronavirus 2 (SARS-CoV-2) spike antibody titers in serum samples taken from patients pre- and postinfusion with IVIG products; results are presented from all patients (*A*) and according to product received (*B*). Note that 1 patient in the Privigen group had received sotrovimab within the previous 2 weeks and had a titer >25 000 U/mL even before infusion. *C–D*, Comparison of neutralizing antibody titers (Inhibitory Dilution 50 (ID_50_) against SARS-CoV-2 ancestral and Omicron viruses in serum samples from patients pre- and postinfusion with IVIG products. *E–F*, Neutralizing antibody titers in comparison with healthy donors. *P* values were calculated using Wilcoxon paired *t* test for comparing 2 groups and Kruskal-Wallis test with Dunn post hoc test for comparing 3 groups (**P < .01; ***P < .001; ****P < .0001). Abbreviations: HDs, healthy donors; ID_50_, Inhibitory Dilution 50; IVIG, intravenous immunoglobulin; ns, not significant; S Ab, spike antibody.

Most patients showed a significant increase in anti-spike antibody titers postinfusion with immunoglobulin products (Figure 1*A*; *P* < .01) with the median titer increasing from 2123 U/mL preinfusion to 10 600 U/mL postinfusion. Upon classification based on IVIG product administered (Figure 1*B*), this increase in anti-spike antibody titers was observable in most patients receiving Privigen or Octagam. Responses in patients receiving Intratect were variable, perhaps indicating batch-to-batch variation. Postinfusion titers in patients who received Flebogamma were relatively modest, whereas for Iqymune, spike antibody titers remained unchanged or decreased slightly and some were very low (<100 U/mL). The patient who had received sotrovimab recently had a titer of >25 000 U/mL even pre-IVIG infusion (receiving Privigen). Three patients receiving Intratect had similarly high titers preinfusion even in the absence of recent treatment.

Pseudovirus neutralization titers against both ancestral and Omicron BA.1 variants significantly increased in sera postinfusion with IVIG products (Figure 1*C* and 1*D*). Baseline pseudovirus neutralization titers against ancestral spike tended to be higher than Omicron prior to infusion with IVIG products (median ID_50_, 1437 vs 925).

We also compared the SARS-CoV-2 neutralization titers of patients with immunodeficiencies undergoing IVIG infusions to vaccinated healthy individuals. Pseudovirus neutralizing antibody titers against both ancestral and Omicron viruses were significantly lower in patients prior to infusion with IVIG products compared to healthy donors (median ID_50_: ancestral, 1437 [95% confidence interval {CI}, 913–2141] vs 27 574 [95% CI, 3538–70 474], *P* < .0001; Omicron, 925 [95% CI, 544–2216] vs 7563 [95% CI, 6047–14 936], *P* < .001) but were restored to a median level comparable to those observed in healthy donors after infusion, albeit with considerable heterogeneity (median ID_50_: ancestral, 4985 [95% CI, 2964–9682] vs 27 574 [95% CI, 3538–70 474]; Omicron, 4932 [95% CI, 3436–6690] vs 7563 [95% CI, 6047–14 936]; no statistically significant differences) (Figure 1*E* and 1*F*). There were no statistical differences between spike antibody concentrations or neutralization titers (pre- or postinfusion) between patients with a history of COVID-19 in the last 6 months versus those who had COVID-19 >6 months previously.

### IVIG Products Exhibit SARS-CoV-2 Neutralization Capacity but With Differences Between Products and Viral Variants

To confirm that neutralization was derived from the immunoglobulin products, we tested some of the corresponding batches of IVIG products (except Gammaplex) used on those days in the clinic at neat concentration followed by 5-fold serial dilutions in the pseudovirus neutralization assay. All products demonstrated detectable neutralization varying between ID_50_ of 1:25 000 and 1:2 × 10^7^, higher titers overall than observed in patient serum. Batches of Intratect and Privigen showed significantly higher neutralization activity against ancestral virus compared to other products, especially Flebogamma and Iqymune, which is in line with spike antibody results observed previously (Supplementary Figure 1*A*).

To ascertain whether these differences between IVIG products are reflected in the neutralizing antibody titers of patient serum, postinfusion neutralization titers were compared based on the IVIG product received. Lower neutralization against ancestral virus was seen in serum from patients who received Iqymune, consistent with other results. However, a high degree of patient-dependent variability in neutralization titers was observed in our cohort (Supplementary Figure 1*B*). This is likely to reflect varying immunological outcomes from prior vaccination or infection, with some patients able to generate an antibody response and others not. Nevertheless, the overall improvement in neutralization across the cohort from pre- to postinfusion (Figure 1) suggests that most IVIG products are conferring additional benefit, even against Omicron BA.1.

**Figure 2.**
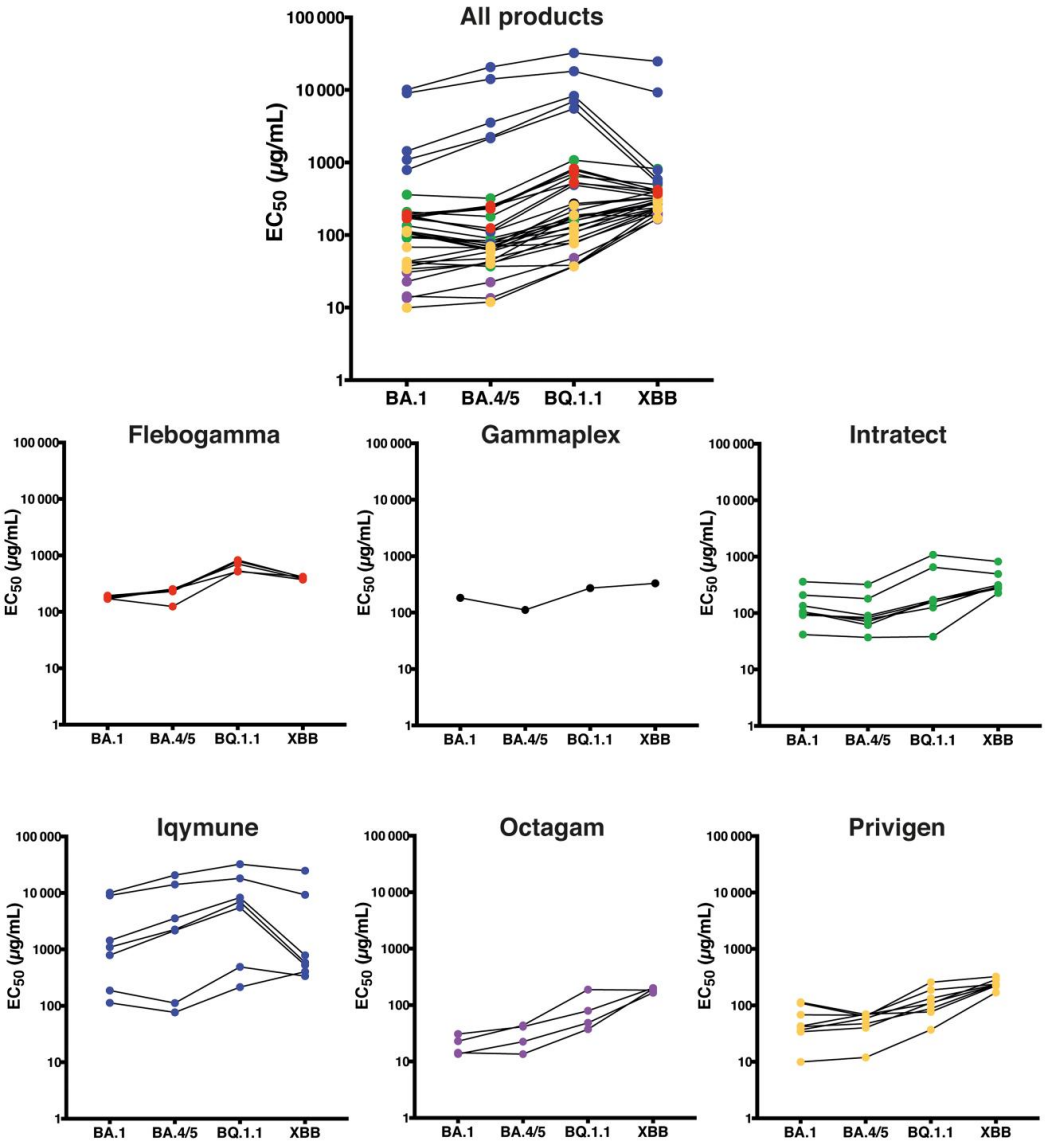
Intravenous immunoglobulin (IVIG) products have neutralizing activity against circulating variants BQ.1.1 and XBB. Batches of 6 different IVIG products were assessed using a high-throughput live-virus microneutralization assay against Omicron BA.1 and subvariants, including BA.4/5, BQ.1.1, and XBB. The half maximal effective concentration (EC_50_) values were calculated fitting a 4-parameter dose-response curve to 4 replicate runs of an 8-point dilution series of each product (Supplementary Figure 3) and represents the concentration of the product (µg/mL) that effectively inhibits viral infection and replication by 50%.

To further interrogate the potential effect on current Omicron subvariants, we analyzed immunoglobulin products with a live-virus microneutralization assay against Omicron BA.1, BA.4/5, BQ.1.1, and XBB variants. Batches tested in these experiments include some of those used for earlier assays plus some additional vials collected more recently. Results confirm differences between products and between batches with significant heterogeneity for some (eg Intratect) and generally poorer neutralization for Iqymune compared to other products (although 2 batches demonstrated better neutralization, which might reflect more recent plasma harvest) (Figure 2). Results were broadly in line with spike antibody titers measured in patient serum (Figure 1*A*). Interestingly, neutralization of BA.4/5 variants appeared somewhat better on average than for BA.1.

Privigen and Octagam, which had the lowest half maximal effective concentration (EC_50_) for earlier Omicron variants, tended to neutralize BQ.1.1 and especially XBB relatively less effectively, but at a level similar to other products. Interestingly, when products have better neutralization against BA.4/5 than against BA.1, these products neutralize BQ.1.1 somewhat better than XBB, and the inverse also seems true (eg, the less effective Iqymune batches neutralize BA.1 and XBB better than BA.5 and BQ.1.1, whereas the more effective 2 batches neutralize BA.5 better than BA.1 and have slightly better neutralization against BQ.1.1 than XBB; Figure 2). This is in line with the fact that BQ.1.1 is a BA.5 sublineage, whereas XBB is a recombinant of BA.2.75 and BJ.1.

### Commencing IVIG During COVID-19 Disease in Patients With Failure of Humoral Immunity to SARS-CoV-2 May Help Viral Clearance

**Figure 3.**
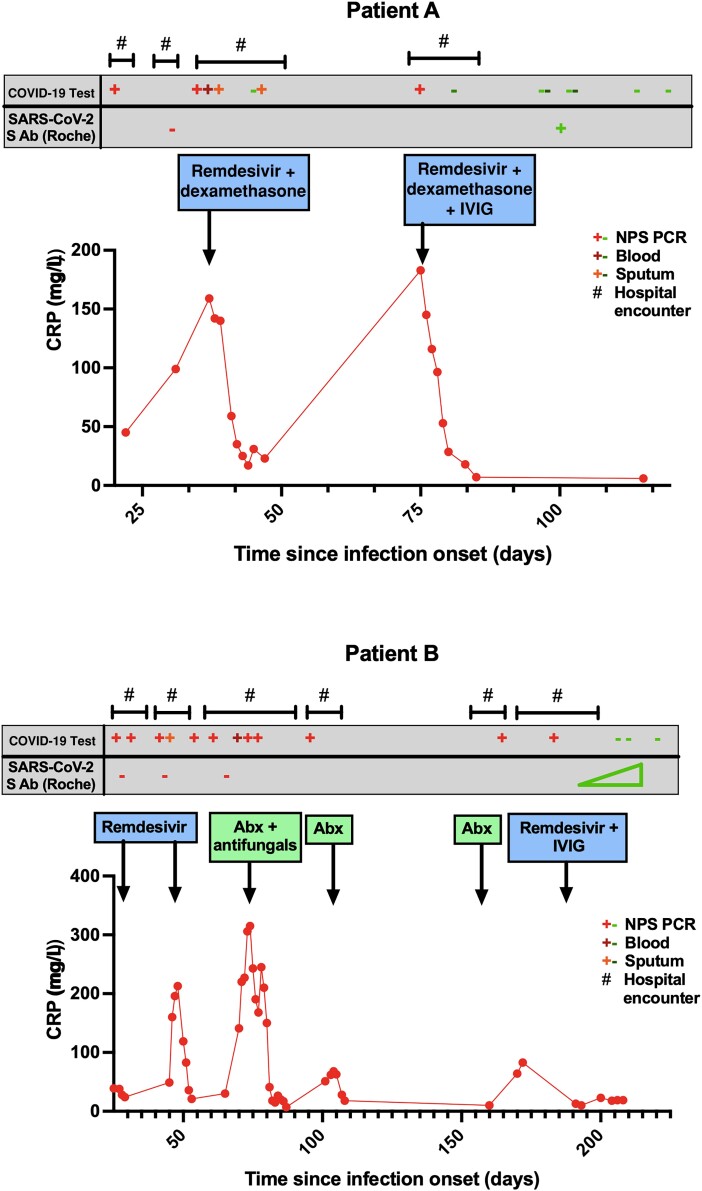
C-reactive protein, severe acute respiratory syndrome coronavirus 2 diagnostic tests, and serum spike antibody results over time during prolonged coronavirus disease 2019 infection with treatment intervention. Abbreviations: Abx, antibiotics; COVID-19, coronavirus disease 2019; CRP, C-reactive protein; IVIG, intravenous immunoglobulin; NPS, nasopharyngeal swab; PCR, polymerase chain reaction; SARS-CoV-2, severe acute respiratory syndrome coronavirus 2; S Ab, spike antibody.

We hypothesized that patients with insufficient SARS-CoV-2 spike antibody who commenced IVIG during a symptomatic COVID-19 illness might achieve viral clearance. Nine patients were started on IVIG (for secondary hypogammaglobulinemia with failure of vaccine response; see Table 2) plus remdesivir in the context of COVID-19 disease, including several with a chronic and relapsing course (Table 2). Some patients had received multiple courses of remdesivir monotherapy, which tended to reduce C-reactive protein (see representative clinical case histories in Figure 3) but did not clear the virus, and the patients suffered further clinical relapse. Median disease duration prior to commencement of IVIG was 189 (range, 23–901) days; median time from IVIG to the last positive PCR result was 13 (range, −5 to 59) days and to the first consistently negative PCR result was 20 (range, 7–81) days.

**Table 2.** Clinical Details of Patients Treated With Intravenous Immunoglobulin as Part of Coronavirus Disease 2019 Therapy

Patient	Age Category, y	Sex	Diagnosis	Anti-CD20	Date of Infection Onset^a^	Lineage	Days Postinfection Onset With COVID-19–Compatible Chest Imaging	IVIG Product	No. of Days From Onset to IVIG Treatment	S Ab Before IVIG (Roche Assay, U/mL)	No. of Days From Onset to S Ab Measurement, Pre-IVIG Treatment	S Ab After IVIG (Roche Assay, U/mL)	Baseline IgG (NR 7–16 g/L)	Baseline IgA (NR 0.7–4 g/L)	Baseline IgM (NR 0.4–2.3 g/L)	B-Cell Count (NR 0.1–0.5 × 10^9^/L)	No. of Days Postinfection Onset of Final Positive Test	No. of Days Postinfection Onset of Consistently Negative Tests	No. of Negative Results	No. of Courses of Remdesivir	Notes
A	65–69	F	Follicular lymphoma	Yes	Jun-22	BA.5	15, 22, 31, 38, 75, 76, 79	Privigen	80	<0.4	78	2209	3.7	0.8	0.2	0.001	75	95	7	2	…
B	70–74	F	Follicular lymphoma	Yes	Mar-22	BA.2.10	25, 48, 71, 75, 76, 101, 192	Kiovig	195	30.2	193	1886	2.4	0.2	0.1	0.14	191	206	3	3	…
C	80–84	F	MALT lymphoma	Yes	Sep-22	BA.5.1	12	Octagam	23	94	21	>2500	4.2	0.3	0.1	0.004	41	63	7	1	Only 1 dose of 0.4 mg/kg IVIG received as part of initial therapy
D	70–74	F	Rheumatoid arthritis	Yes	Aug-22	BE.1.1	25, 35, 40, 53, 56, 68, 77, 79, 84, 91	Privigen	63	<0.4	61	>2500	3.8	1.6	0.2	0.002	77	89	3	2	…
E	65–69	M	Follicular lymphoma	Yes	Mar-22	BA.5.2	0, 194, 198, 199	Octagam	199	<0.4	195	>2500	4.4	0.2	<0.1	0	237	258	6	1	Negative NPS and sputum at day 218 but positive again at day 237, persistently negative from day 258 onward
F	75–79	M	CLL	No	Mar-22	BA.2	56, 262	Privigen	263	9.4	263	>2500	2.7	0.2	<0.1	N/A (B-CLL)	268	276	3	1	Negative tests at days 276, 279, and 282 but positive again at days 298 and 300. Treated with nirmatrelvir/ritonavir. Negative again from day 318.
G	50–54	F	Rheumatoid arthritis	Yes	Jun-22	B.31	15, 16, 21, 63, 176, 304, 353, 388, 600, 696, 757, 771, 849, 866, 867, 868, 878, 885, 889, 893, 898	Octagam	901	<0.4	899	>2500	19.5 *	0.4	<0.1	0.001	960	982	3	1	* Paraprotein 14 g/L. Negative tests at day 913 and 917 but positive again day 920 and 942, negative NPS days 948, 959, and 960, but sputum positive on day 960, negative consistently from day 982
H	60–64	F	Follicular lymphoma	Yes	Jun-22	BA.2	134, 176, 179	Privigen	189	<0.4	189	>2500	3.5	0.9	<0.1	0.001	186	196	4	1	Initially treated with Paxlovid in May/June 2022; only received 1 dose of IVIG as part of initial therapy
I	75–79	M	Follicular lymphoma	Yes	Nov-22	BQ.1.1.13	30, 41, 45	Privigen	55	<0.4	47	>2500	5.2	0.4	0.1	0	68	75	4	1	…

Abbreviations: CLL, chronic lymphocytic leukemia; COVID-19, coronavirus disease 2019; F, female; IgA, immunoglobulin A; IgG, immunoglobulin G; IgM, immunoglobulin M; IVIG, intravenous immunoglobulin; M, male; MALT, mucosa-associated lymphoid tissue; NPS, nasopharyngeal swab; N/A, not applicable; S Ab, spike antibody.

Date of infection onset as per onset of patient symptoms, imaging consistent with COVID-19 and/or positive test result.

Before IVIG treatment, all patients had hypogammaglobulinemia (or effective hypogammaglobulinemia due to paraprotein in 1 patient) and negative or low levels of SARS-CoV-2 spike antibody (Table 2). One patient had B-cell chronic lymphocytic leukemia while all but 1 of the other patients had low levels of peripheral blood B cells. IVIG (Octagam, Privigen, or Kiovig) was administered at replacement doses of 0.5 g/kg. In all but 2 cases, the first 2 doses were given on consecutive days. IVIG was then given approximately once every 4 weeks.

Serum spike antibody levels rose significantly following the initial infusions and all patients subsequently cleared the virus, albeit PCR remained positive for a variable period after treatment (median time from IVIG initiation to the first of consecutively negative tests was 20 days). One patient had a reappearance of positive PCR tests following 3 negative tests and, although asymptomatic, was treated with additional nirmatrelvir/ritonavir before achieving sustained negative tests. Sequencing suggested that this was a recrudescence of the previous virus rather than reinfection. Patient A, who was the first to be treated, has had follow-up computed tomographic (CT) imaging of the chest, which demonstrated significant improvement (Supplementary Figure 2).

Viral sequencing generally revealed Omicron variants, consistent with the timing of onset of illness in most patients. However, sequencing of 2 separate isolates indicated that patient G, diagnosed in December 2022, was infected with a B.31 (clade 19A) strain, last seen in the United Kingdom in mid-2020. This patient had myeloma and rheumatoid arthritis on anti-CD20 therapy and had suffered an undiagnosed respiratory illness with ground-glass changes on CT chest imaging since June 2020. The duration of illness was therefore at least 901 days before treatment. Since commencing IVIG, the patient has experienced significant clinical improvement, has been discharged from hospital, and now has had serial negative SARS-CoV-2 PCR tests on nasopharyngeal swabs.

## DISCUSSION

Patients with antibody deficiency had poorer outcomes from COVID-19 early in the pandemic [4]. Congenital absence or iatrogenic depletion of B cells has emerged as a particular risk for poor outcome [11, 12], prolonged infection [3] and failure of vaccination response [13]. However, more recent data have indicated that the situation is improving in cohorts under the care of clinical immunology services [14]. While this will partly relate to the lower severity of illness with Omicron variants, and may reflect a protective response to multiple vaccinations (including a T-cell response [2, 15]), it may also indicate a protective effect of IRT.

We have demonstrated here that, from mid to late 2022, most commercial IVIG preparations contained neutralizing anti–SARS-CoV-2 spike antibodies and that these are detectable in patient serum after infusion of “replacement” doses (generally 0.4–0.6 g/kg). Most people have levels of spike antibody after infusion that would have rendered them ineligible for antibody-based treatments under some guidelines [16]. They would be considered spike antibody positive in the lateral flow immunoassay devices used in at-home testing studies [17]. Although antibody-deficient patients had lower pseudovirus neutralization capacity than healthy controls immediately preinfusion (at IgG trough), this is largely restored by infusion. Accordingly, although we note that many of our patients had previously had COVID-19, all had made a full clinical recovery.

Testing products directly, including in a live-virus neutralization assay and with current Omicron variants, confirmed neutralization capacity. However, there was variability between products and, especially for Iqymune and Intratect, between batches. This presumably relates to the timing of plasma harvest and is likely to improve over time. Importantly, neutralization against newer Omicron variants (BQ.1.1 and particularly XBB) was broadly retained despite their relative immune escape [18, 19] (albeit the EC_50_ tended to be higher for XBB compared to BA.1 or BA.4/5 for Octagam and Privigen, which otherwise demonstrated the greatest neutralization of earlier variants).

Unlike previous “waves” dominated by specific variants of concern, multiple Omicron subvariants, including BQ.1.1 and XBB, are co-circulating through populations. Differences in batches and products thus reflect not only geographical and temporal differences in plasma collection, but potentially differential subpopulational exposures. Nevertheless, the retention of neutralizing capacity against current variants despite plasma harvest at least several months ago implies that donors have generated broadly neutralizing responses to vaccination or prior infection.

We then examined administration of IVIG during treatment of COVID-19. Eligibility for immunoglobulin administration in the United Kingdom is according to strict criteria, and all patients included here were hypogammaglobulinemic (or functionally so due to presence of paraprotein) with severe or recurrent infection and failure of SARS-CoV-2 antibody response despite vaccination and prolonged illness. Most patients had absent B cells, consistent with previous reports [3, 20, 21]. Several had received previous remdesivir monotherapy but relapsed, as we have previously observed [3]. All patients recovered clinically with viral clearance at a median of 20 days. In some cases, tests became negative but then positive again and in 1 case the patient was treated with additional nirmatrelvir/ritonavir. This suggests that, although IVIG may be a useful adjunctive treatment, patients need to be monitored closely for viral clearance. There is a risk of intrahost evolution during prolonged infection [22], and partially effective treatments may contribute to this [23–25]. Polyclonal products such as IVIG may have advantages over monoclonal antibodies in terms of multiple target epitopes and a broader mechanism of action: however, a wide range of lower concentration antibodies may also select for escape mutants if infection does not clear rapidly. Given the variability between products and batches, testing the neutralization capacity of a batch may be prudent before use as part of therapy. As a minimum, clinicians should check that the anti–SARS-CoV-2 spike antibody titer in serum has risen significantly postinfusion.

Our results are also consistent with the results of studies using convalescent plasma, which appears to confer a survival advantage when used to treat COVID-19 in immunocompromised patients [6, 7].

In our center, IVIG was always given in combination with remdesivir, but data from clinical trials are lacking and it is unknown whether combination therapy is definitively required for chronic SARS-CoV-2 infection. However, we note that many patients in our center had persistent SARS-CoV-2 PCR positivity beyond 28 days despite IRT and often with other treatment interventions [26], while persistence after imdevimab/casirivimab was also seen [26]. Others have also suggested the need for combinations when administering neutralizing monoclonal antibodies for COVID-19 to this patient population [25].

One patient in our series had a B.31 ancestral viral variant with persistent respiratory illness and radiological abnormalities from June 2020 until December 2022. To our knowledge, this is longer than any case reported previously [27]. This case highlights the need for a high index of suspicion of chronic COVID-19 in highly immunosuppressed patients and the need for serial testing if the clinical presentation is consistent. Further sequencing is under way to characterize the extent of viral mutation over the infection period of 2.5 years.

Our study has limitations. We were not able to investigate every IVIG product as our center does not use all available preparations. We have also not investigated subcutaneous immunoglobulin products, although we anticipate that findings would be similar. The pseudovirus neutralization assay only used a BA.1 Omicron variant, but the live-virus tests on the products utilized current circulating variants. Some patients in the cross-sectional observational study may have mounted an antibody response to vaccination and this was not possible to assess independently. IVIG was given to patients with COVID-19 as part of clinical care and not as a formal clinical trial, and thus there were some differences in protocol and sample collection between patients.

In summary, we have demonstrated that immunoglobulin products now contain important SARS-CoV-2 antibodies and that these are transmitted to patients and may be useful in protection against severe or persistent infection. IVIG administration during COVID-19 disease appears to help viral clearance. However, further studies, and ideally randomized trials, are required to identify the optimal treatment in patients at highest risk of persistent infection or with established chronic COVID-19.

## Supplementary Data

Supplementary materials are available at *Clinical Infectious Diseases* online. Consisting of data provided by the authors to benefit the reader, the posted materials are not copyedited and are the sole responsibility of the authors, so questions or comments should be addressed to the corresponding author.

## Supplementary Material

ciad368_Supplementary_Data

## Notes

***Author Contributions.*** D. M. L. conceived the study. K. T., M. O., F. M., S. W., and A. S. recruited patients for the cross-sectional study and collected data and samples. S. S., D. S., S. F. K. L., N. C., J. M. G., L. N., S. H., and S. R. provided clinical care to patients with COVID-19 and collected clinical data and samples. A. T. provided pharmacy oversight. J.. C. L. H. provided diagnostic virology data and samples. L. M., V. U., and M. Y. W. designed experiments. V. U., M. Y. W., A. H., G. D., M. R., and L. S. H. performed experiments. E. J. C. and M. Y. W. analyzed the live-virus microneutralization experiments. S. O. B., S. T., L. M., R. C. L. B., E. C. W., and D. M. L. provided intellectual input. V. U., K. T., M. Y. W., and D. M. L. wrote the manuscript. All authors reviewed and approved the manuscript.

***Acknowledgments.*** The authors acknowledge the generosity of the patients reported here. The authors would like to thank Rosemarie Ford and Dylan Jankovic of the Institute of Immunity and Transplantation, University College London (UCL), for their technical input with the pseudovirus neutralization assay.

***Financial support.*** R. C. L. B. reports intramural funding from the Francis Crick Institute.
