# Supplementary material for: Commercial Immunoglobulin Products Contain Neutralizing Antibodies Against Severe Acute Respiratory Syndrome Coronavirus 2 Spike Protein
Source: Clin Infect Dis. 2023 Jun 20;77(7):950–60. doi: 10.1093/cid/ciad368 (PMC10552578; doi:10.1093/cid/ciad368)

**Commercial immunoglobulin products contain neutralising antibodies against SARS-CoV-2 spike protein**

**Supplementary Material**

**Supplementary Table 1: Virus lineages used in the live-virus microneutralisation assay.**

| **Lineage** | **Mutations present in Spike** | **Source** |
| --- | --- | --- |
| Omicron BA.1  [M21021166] | A67V, Δ69-70, T95I, Δ142-144, Y145D, Δ211, L212I, G339D, S371L, S373P, S375F, K417N, N440K, G446S, S477N, T478K, E484A, Q493R, G496S, Q498R, N501Y, Y505H, T547K, D614G, H655Y, N679K, P681H, A701V, N764K, D796Y, N856K, Q954H, N969K, and L981F | Prof. Gavin Screaton, University of Oxford, Oxford, UK via the Genotype-to-Phenotype National Virology Consortium (G2P-UK) |
| Omicron BA.5 | T19I, Δ24- 26, A27S, Δ69-70, G142D, V213G, G339D, S371F, S373P, S375F, T376A, D405N, R408S, K417N, N440K, L452R, S477N, T478K, E484A, F486V, Q498R, N501Y, Y505H, D614G, H655Y, N679K, P681H, N764K, D796Y, Q954H, and N969K | Prof. Alex Sigel, Prof. Tulio de Oliveira, Africa Health Research Insitute, Durban, South Africa via the Genotype-to-Phenotype National Virology Consirtium (G2P-UK) |
| Omicron XBB  [hCoV/England/FCI-191/2022] | T19I, L24-, P25-, P26-, A27S, V83A, G142D, Y144-, H146Q, Q183E, V213E, G339H, R346T, L368I, S371F, S373P, S375F, T376A, D405N, R408S, K417N, N440K, V445P, G446S, N460K, S477N, T478K, E484A, F486S, F490S, Q498R, N501Y, Y505H, D614G, H655Y, N679K, P681H, N764K, D796Y, Q954H, N969K | Francis Crick Institute |
| Omicron BQ.1.1  [hCoV/England/FCI-190/2022] | T19I, L24-, P25-, P26-, A27S, H69-, V70-, V213G, G339D, R346T, S371F, S373P, S375F, T376A, D405N, R408S, K417N, N440K, K444TL452R, N460K, S477N, T478K, E484A, F486V, Q498R, N501Y, Y505H, D614G, H655Y, N679K, P681H, N764K, D796Y, Q954H, N969K | Francis Crick Institute |

**Supplementary Figure 1: (A)** Product wise comparison of neutralising antibody titres (ID_50_) against SARS-CoV-2 Ancestral virus in neat IVIG products used for infusions in patients. **(B)** Neutralising antibody titres (ID_50_) against Ancestral Spike bearing pseudovirus in patient serum post-infusion with IVIG products. *P*-values were calculated using non-parametric Mann-Whitney test (**P*<0.05; ***P*<0.01).


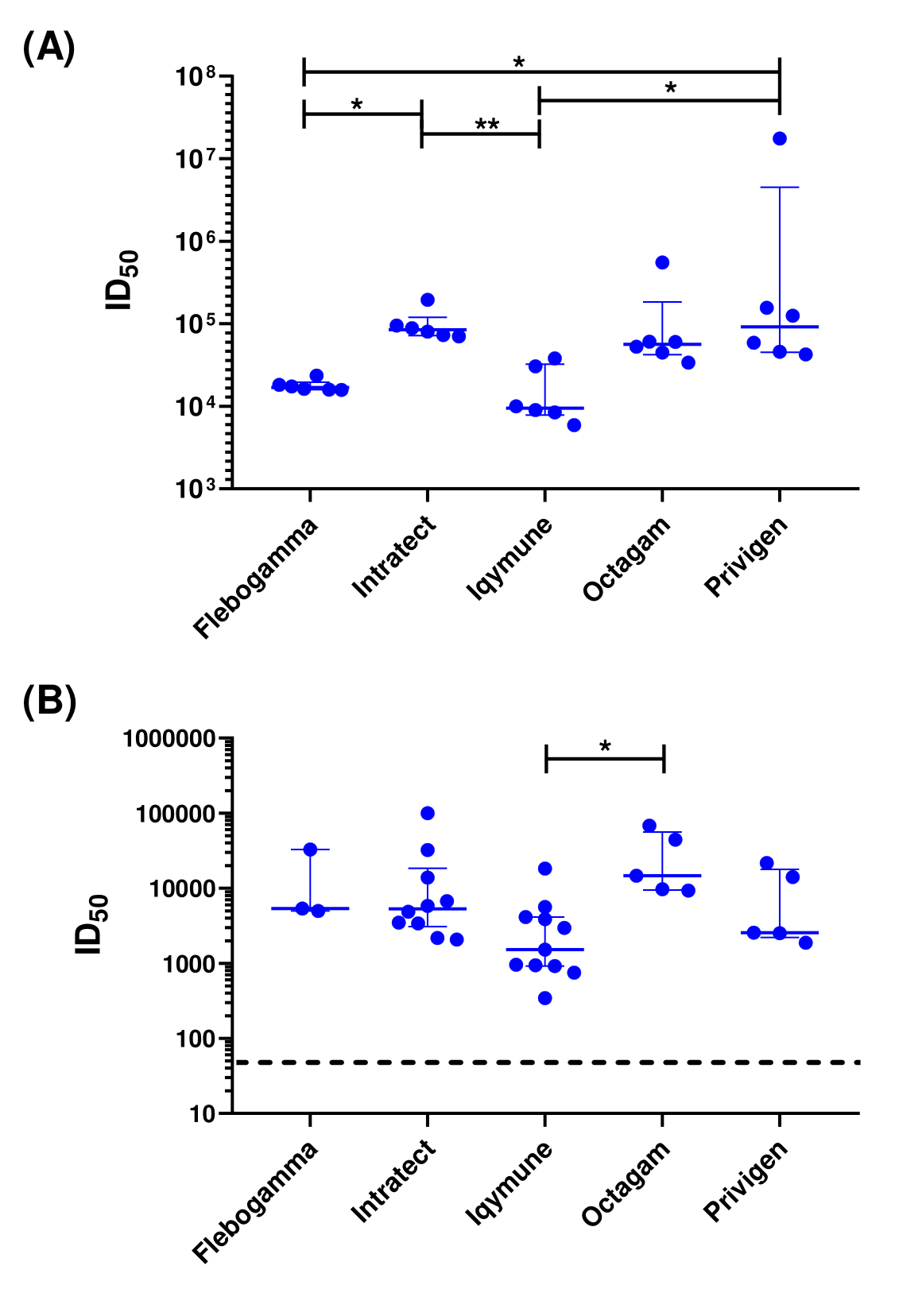


**Supplementary Figure 2. CT chest imaging over course of infection in Patient A.**

| Day 30 of COVID-19 infection |  |
| --- | --- |
| 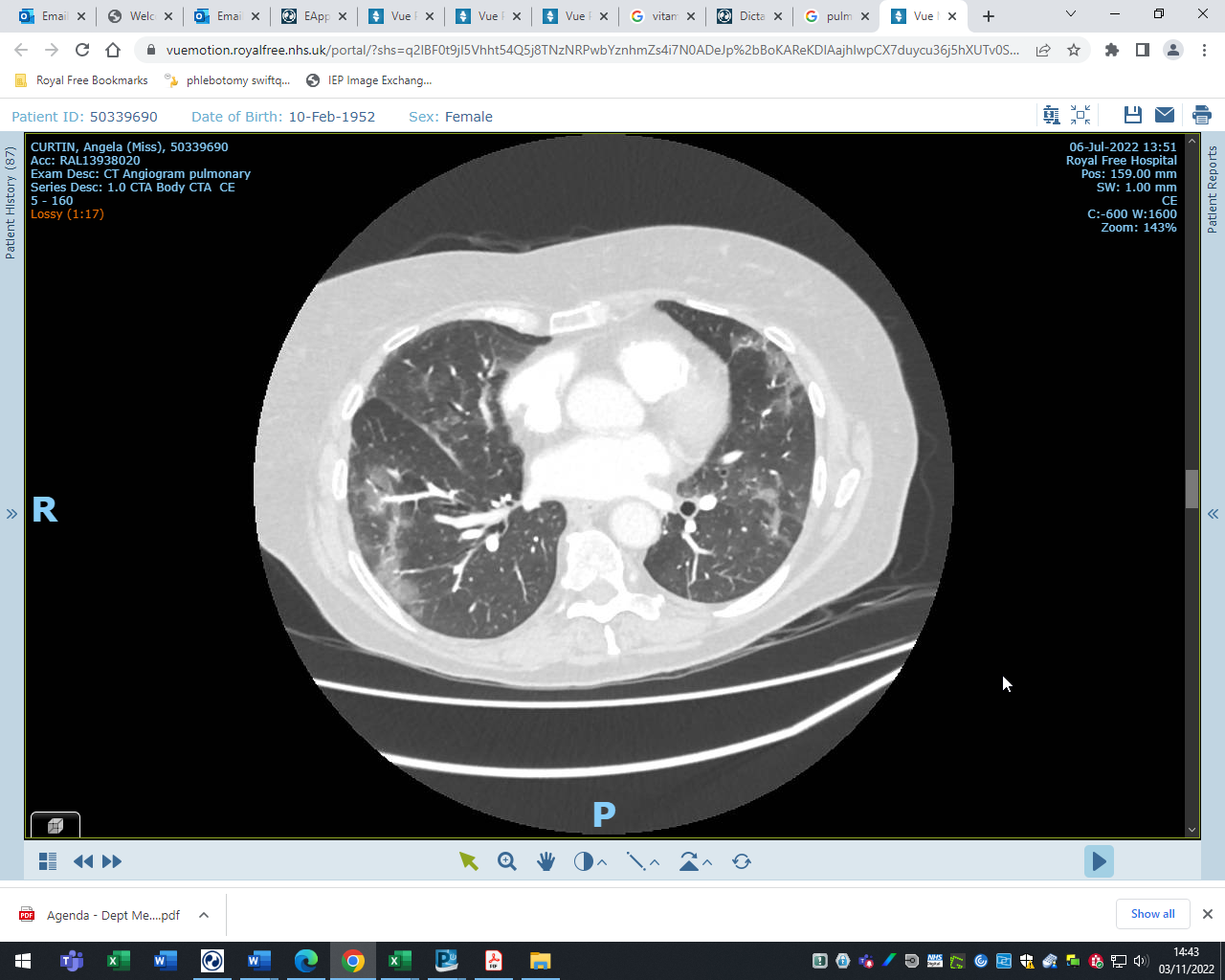 | Multifocal ground-glass throughout both lungs suggestive of COVID-19 pneumonitis |
| Day 75 of COVID-19 infection |  |
| 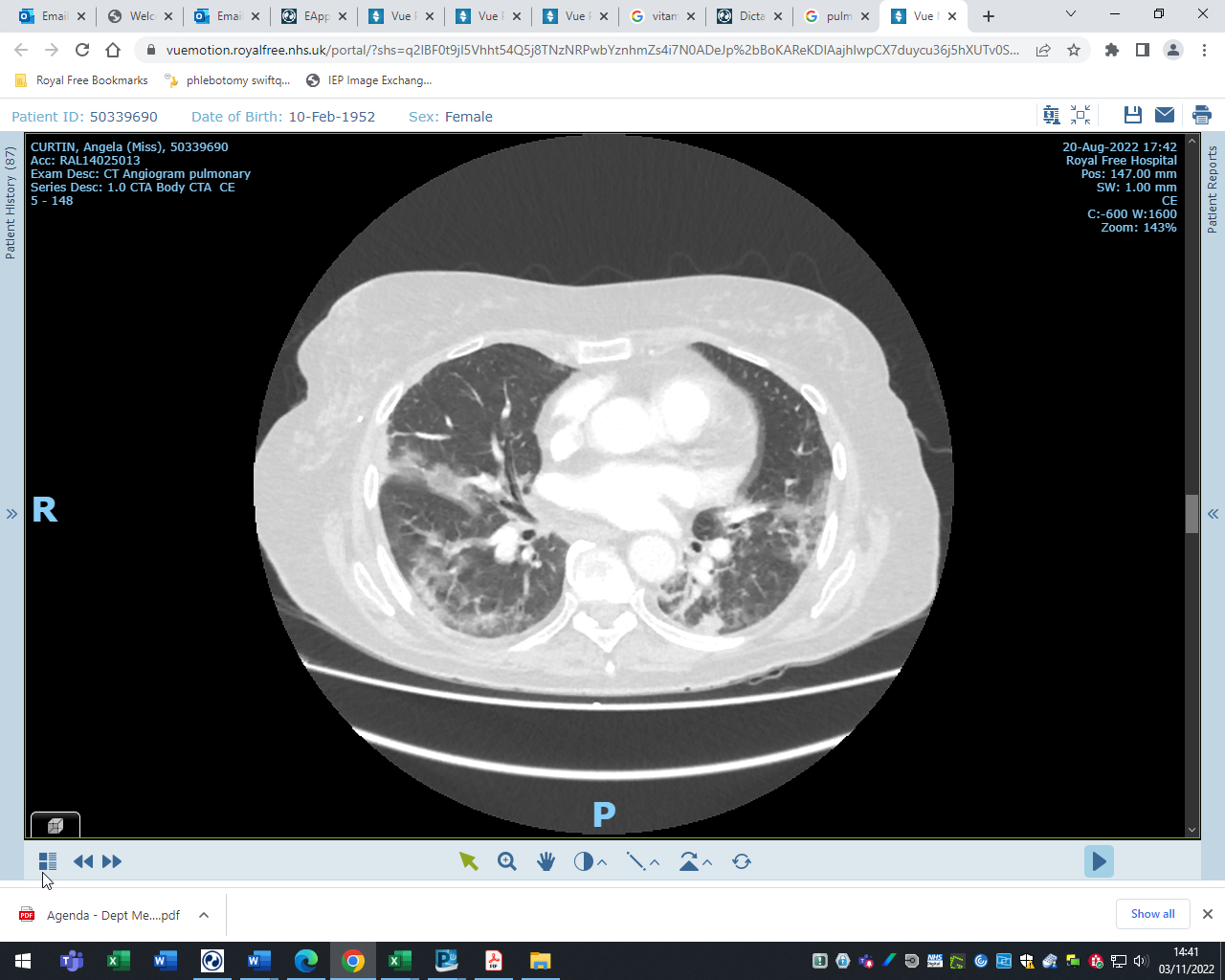 | Diffuse bilateral ground-glass opacification/ consolidation predominately in the lower lobes and right upper lobe. Appearances have progressed since the last CT scan. |
| Day 80 after receiving IVIG |  |
| 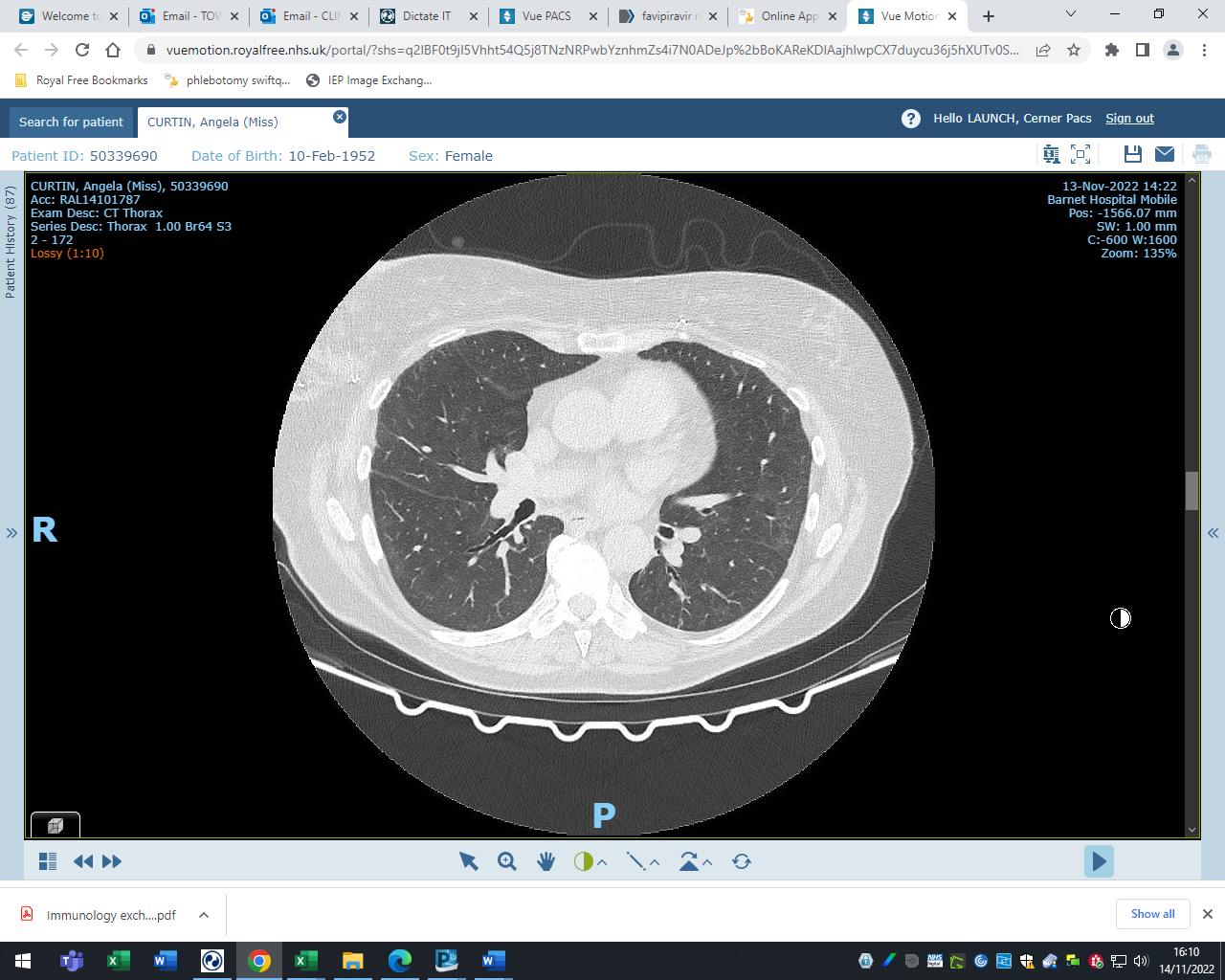 | Resolution in bilateral opacifications. |

**Supplementary Figure 3. Dose-response curves fitted to live-virus microneutralisation data for EC50 estimation.**


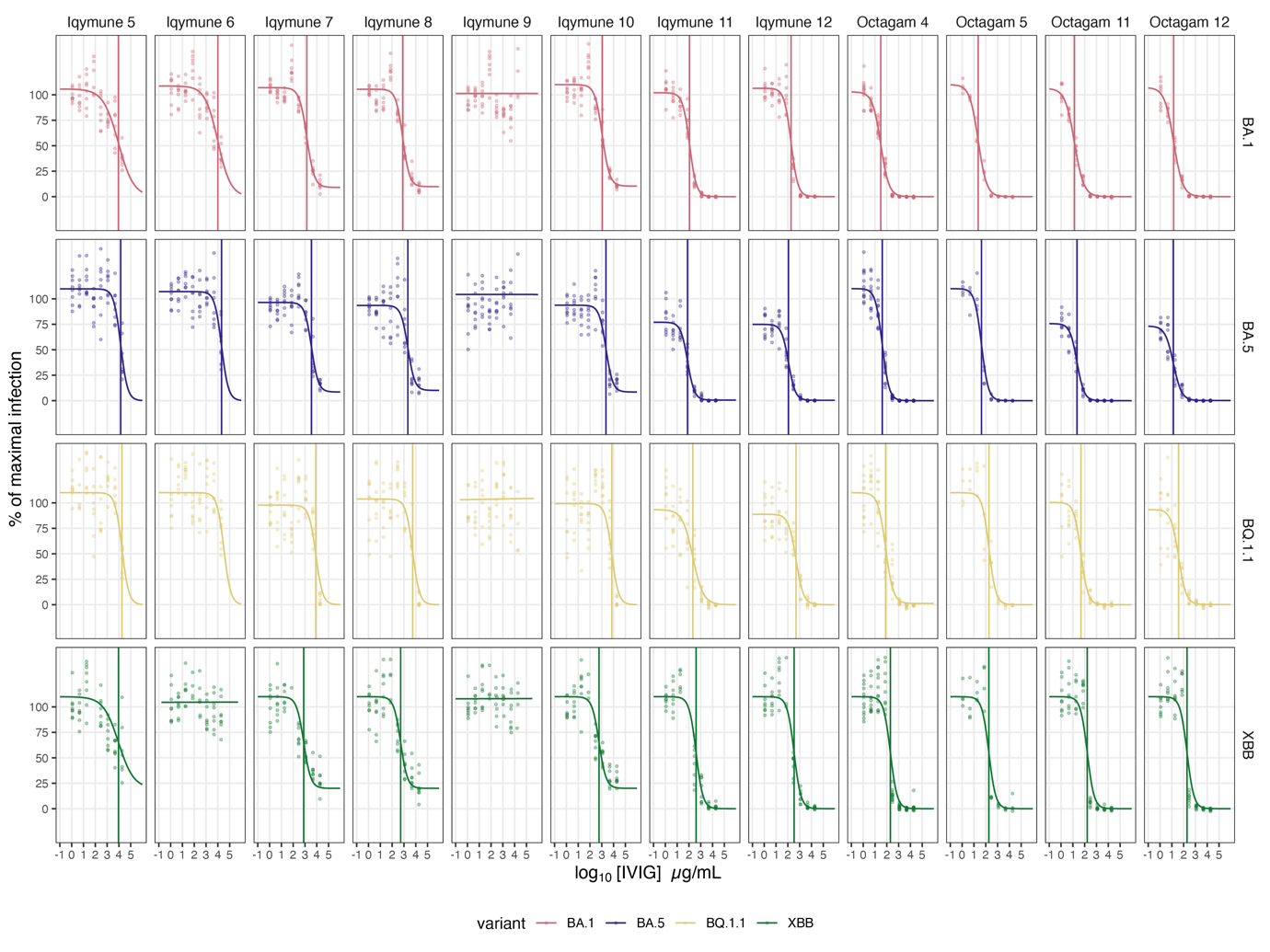

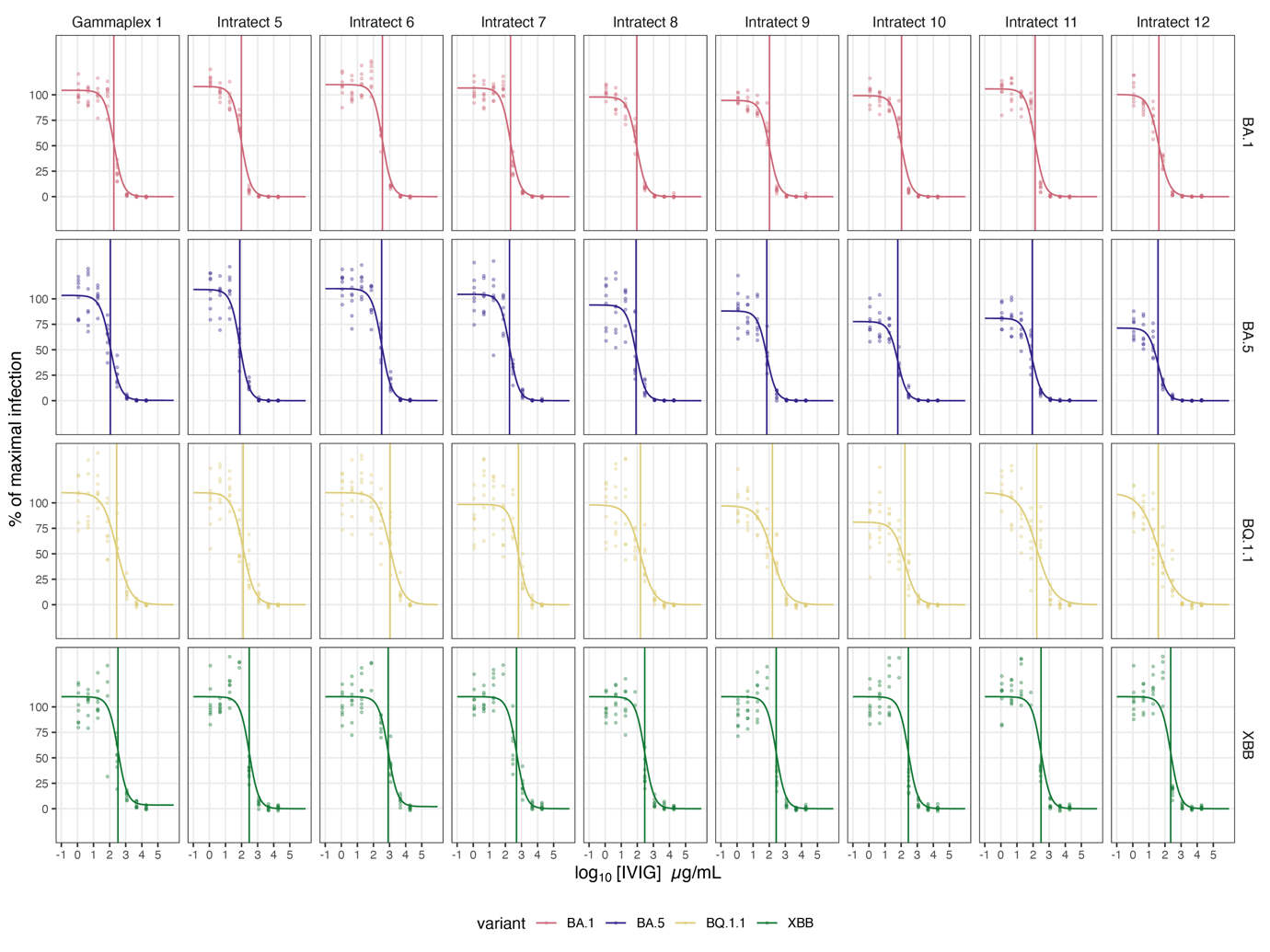


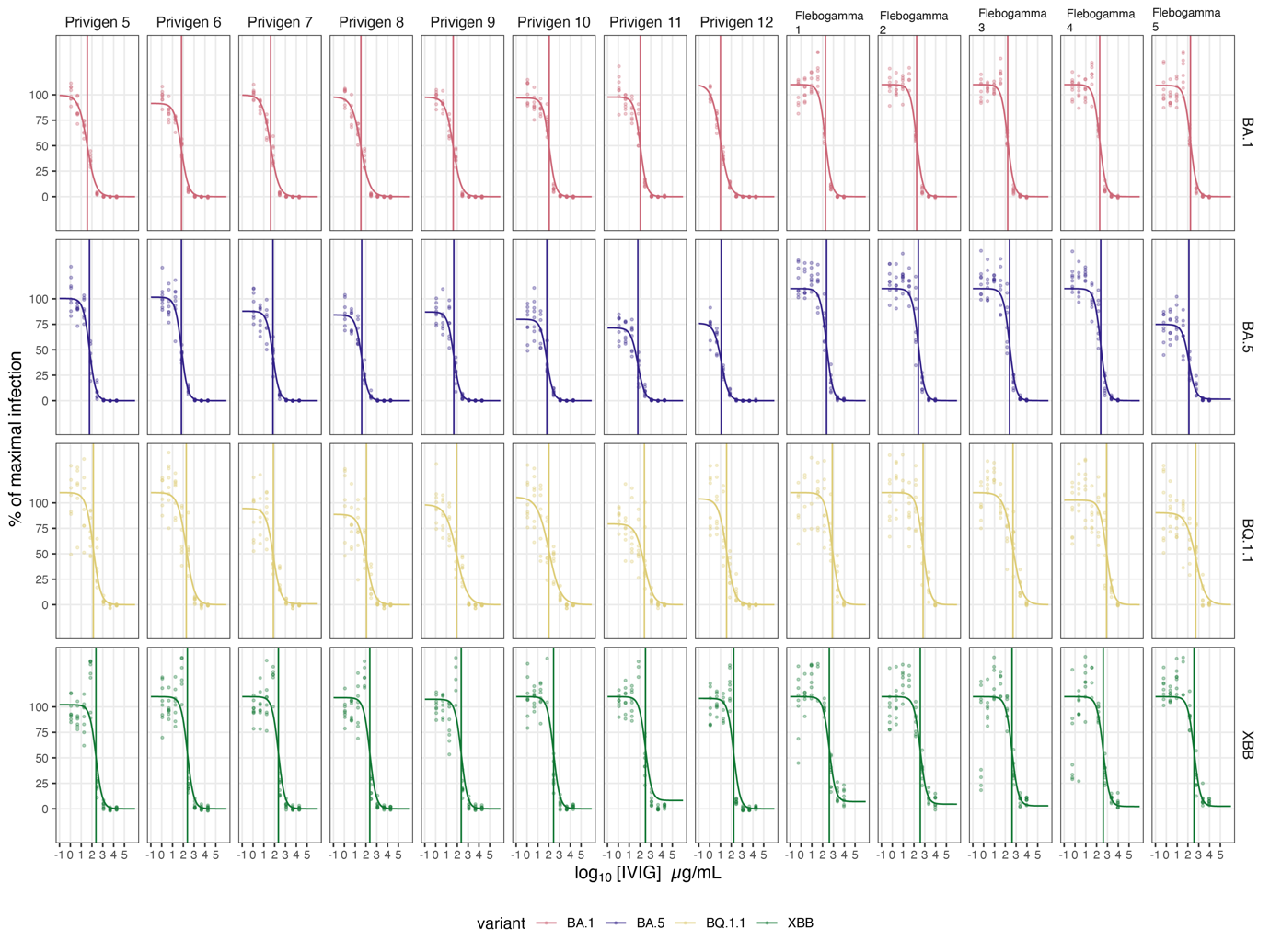

Supplement: ciad368_Supplementary_Data [file ciad368_supplementary_data.docx]
